# Association of hypertension with helicobacter pylori: A systematic review and meta‑analysis

**DOI:** 10.1371/journal.pone.0268686

**Published:** 2022-05-19

**Authors:** Yizhen Fang, Huabin Xie, Chunming Fan

**Affiliations:** 1 Department of Clinical Laboratory, School of Medicine, Xiamen Cardiovascular Hospital of Xiamen University, Xiamen University, Xiamen, China; 2 Xiamen Key Laboratory of Precision Medicine for Cardiovascular Disease, Xiamen, China; Nitte University, INDIA

## Abstract

**Background and aims:**

The number of hypertensive population rises year by year recently, and their age becomes more youthful. For a long time, hypertension has long been regarded as a multi-factorial disease. In addition to smoking, genetics, diet and other factors, helicobacter pylori (*H*. *pylori*) had been regarded as a potential risk factor for hypertension in recent years. However, most studies had certain limitations and their results were inconsistent. Thus, it is necessary for us to assess the impact of *H*. *pylori* on hypertension through meta-analysis.

**Methods:**

We searched all published relevant literature through multiple databases by July 23, 2021. Pooled results were calculated under the random effect model. Heterogeneity was evaluated by the Q statistic and the I^2^ statistic. The risk of bias was evaluated via ROBINS-I tool. Publication bias was evaluated by the Egger test and Begg funnel plot.

**Results:**

6 eligible studies involving 11317 hypertensive patients and 12765 controls were selected from 20767 retrieval records. Our research confirmed that *H*. *pylori* significantly increased the probability of suffering from hypertension in the random effect model (OR:1.34, 95% CI:1.10–1.63, P = 0.002, I^2^ = 74%). The same results were also found in both Asian population and developing country (OR:1.28, 95%CI:1.05–1.55, P = 0.003, I^2^ = 78.5%).

**Conclusions:**

Our results confirmed that *H*. *pylori* was a vital risk factor for hypertension. *H*. *pylori-*infected people were 13.4% higher risk for hypertension than uninfected individuals. In addition, it will be a new method to prevent and treat hypertension by eradicating *H*. *pylori*.

**Trial registration:**

The registration number for systematic review in PROSPERO CRD42021279677.

## Introduction

Hypertension is a common senile disease in modern society and is the underlying cause of many cardiovascular diseases such as acute coronary syndrome, aortic dissection, heart failure and aortic dissection. Hypertension remains a global health priority, as twenty-six percent of the population worldwide suffer from hypertension and the percentage is expected to reach twenty-nine percent in five years [1]. According to traditional views, hypertension is considered as a multi-factorial disease which is affected by age, gender, genetic background and living environment [2, 3]. Much of the recent evidence suggests that pathogen infection is a new and important risk factor for hypertension. Vahdat et al found that co-infection of multiple pathogens was significantly related to hypertension [4]. In addition, Mycobacterium tuberculosis infection elevated incidence of hypertension and patients with treated latent tuberculosis infection significantly reduced the risk of hypertension [5]. Several studies have reported that the chronic infection with cytomegalovirus [6], herpes simplex virus [7] and helicobacter pylori [8] was linked with hypertension.

Helicobacter pylori (*H*. *pylori*) is an acid-resistant gastric colonizing bacterium and the extremely high infection rate in the population makes it become the biggest hidden danger to human health. Since *H*. *pylori* was discovered in human stomach, it has long been thought to be only related to gastrointestinal diseases. Currently, many studies have found that *H*. *pylori* was etiologically linked to many extra-intestinal diseases, such as cardiovascular disease, diabetes, immune thrombocytopenic purpura and chronic urticaria [9]. *H*. *pylori* infection could establish lifelong inflammation [10], and persistent low-grade inflammation played an important part in accelerating the progression of hypertension [11]. In addition, high-salt conditions and Vitamin D (Vit D) metabolism close linked to *H*. *pylori* which may be an explanation alternative to hypertension caused by *H*. *pylori* infection [12, 13]. In a multi-center study, systolic blood pressure (SBP) and diastolic blood pressure(DBP) were significantly elevated in *H*. *pylori* positive adults compared with *H*. *pylori* negative adults [14]. A growing body of epidemiological data supported the view that *H*. *pylori* promoted the occurrence of hypertension [8, 15]. Xiong et al reported that *H*. *pylori* was still positively related to hypertension while adjusting for gender, age and family history of hypertension [15]. And beyond that, a 2020 study said that *H*. *pylori* eradication in hypertensive patients could decrease overall mortality and cardiovascular mortality in eastern Asian population [16]. However, Liu et al took a negative opinion on this view [17], and a study in Mongolian population also showed that *H*. *pylori* was entirely unrelated to hypertension [18]. At present, many relevant studies had contradictory results, so the purpose of this study was to explore the etiological correlation between *H*. *pylori* and hypertension, meanwhile, further to determine that whether *H*. *pylori* was a risk factor for hypertension through meta-analysis.

## Materials and methods

We conducted the present review according to the set of items for PRISMA guidelines 2020 [19]. This study protocol has been registered with the PROSPERO(CRD42021279677).

### Literature retrieval strategy

We obtain all published eligible literature from four major databases (PubMed, Cochrane Library, Excerpt Medica Database and Web of Science) by July 23, 2021. The following MeSH (Medical Subject Heading) terms and keywords were used for systematic literature search: (Blood Pressure or Hypertension or hypertensive or high blood pressure or raised blood pressure) and (Helicobacter pylori or Helicobacter or Helicobacter infection or *H*. *pylori* or HP). Language limited to English and the no time limit for publication data.

### Inclusion and exclusion criteria

Two authors conducted literature screening respectively according to the inclusion and exclusion criteria. When there was great divarication in the literature screening process, the third author was required to give a decision.

Inclusion criteria were listed as follows: (1) Study was underwent with the clinical case-control study or cross-sectional study, (2) The exposure factor was *H*. *pylori* infection, (3) the outcome of interest was hypertension, (4) Complete research data was available to calculate odds ratios (ORs) and 95% confidence interval (CIs), (5) Study was carried out on human being.

Exclusion criteria were listed as follows: (1) Some article types including letter, case report, editorial, review and meeting abstract, (2) study participants with a history of helicobacter pylori eradication treatment.

**Fig 1:**
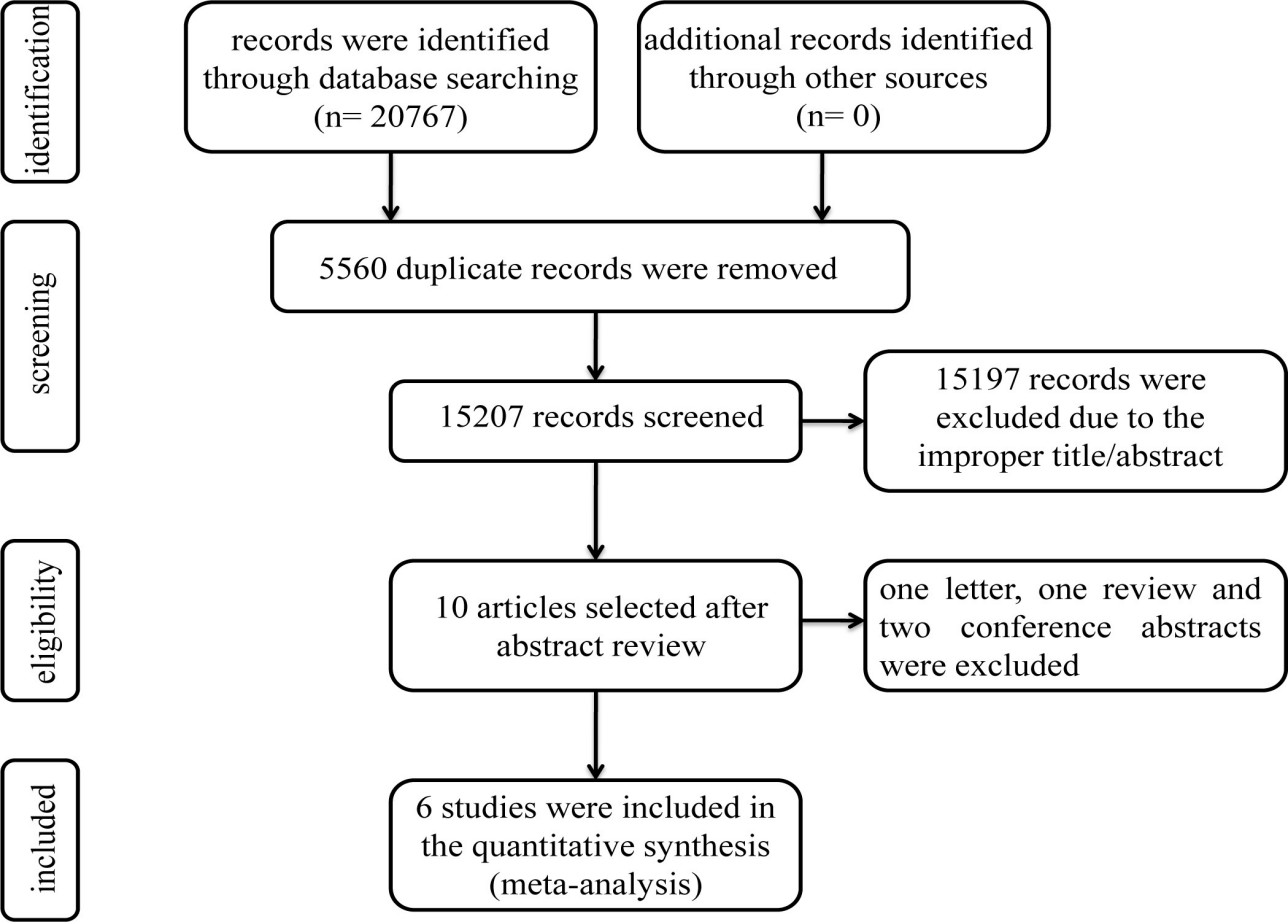
Flow chart of literature screening.

The process of literature screening was showed in the **Fig 1**.

### Data extraction

Two investigators separately obtained data from all included studies. When differences arose, the third investigator was required to give a decision. The extracted data contained the following: the name of the first author, the time of publication, country, race, detection method of *H*. *pylori*, study type, average age of hypertensive patients, human development index (HDI), the number of case and control groups. When there was missing data in the article, our corresponding author would contact with the author to get the missing data.

No automatic tool was used during the whole process.

### Quality score and evaluation of risk of bias

We conducted an exhaustive quality evaluation for each included study according to the Newcastle–Ottawa Scale (NOS) [20]. The NOS describes research with three domains, including selection, comparability and exposure. The quality of included studies were graded from 0 to 9 by the scale and the study with NOS score 7 was perceived as a high quality study. The risk of bias in the included studies was evaluated via ROBINS-I tool (Risk Of Bias In Non-randomized Studies-of Interventions) [21]. The ROBINS-I tool consists of seven domains, including confounding, selection of participants, intervention, deviations from intended interventions, missing data, measurement of outcomes and selection of the reported result. The risk level of bias was divided into five grades: low risk, moderate risk, serious risk, critical risk and no information (NI). Two authors independently evaluated each article according to the NOS and ROBINS-I tool. A third author settle all disputes.

### Statistical analysis

Pooled ORs and 95% CIs were calculated to evaluate the correlation between hypertension and *H*. *pylori*. Heterogeneity was evaluated by the Q statistic and the I^2^ statistic. When the heterogeneity was significant (Q statistic: P<0.1 and I^2^ statistic: I^2^>50%), the pooled ORs and 95% CIs were be calculated under the random effect model. The fixed-effect model was be applied to calculate when statistical results were not remarkable in the Q statistic (P≥0.1). Sensitivity analysis was conducted by eliminating a single study at a time to assess the change in pooled ORs and 95% CIs. Publication bias was evaluated by the Egger test and Begg funnel plot (P<0.05 indicated the result suffer from significant bias). The STATA software V12.0 was applied to statistical analysis in this review.

## Results

### Screening results

A total of 20767 literature were retrieved according to the retrieval strategy. 5560 articles are excluded due to the duplication and 15207 records were further screened by reviewing their titles and abstracts. Then 15197 unrelated studies were removed. Full-text review was conducted in the rest 10 articles and 4 articles were further eliminated, including one letter [22], one review [23] and two conference abstracts [24, 25]. Ultimately, a total of 6 eligible studies with 11317 hypertensive patients and 12765 controls were included in this meta-analysis.

### Major characteristics of included studies

No missing data were found in all included studies and major characteristics of all included studies were showed on **Table 1.** According to the type of study design, three of these studies were cross-sectional studies and the others were case-control studies. Among these studies, one was came from England [8], one from India [26], three from China [15, 17, 27] and one from Italy [28]. The sample size for all studies ranged from 80 to 17100 and studies from China contributed to the largest sample size (98.4%). In three Chinese studies, the control group included volunteers with normal blood pressure from the community and non-hypertensive patients form hospital. However, three other studies from England, India and Italy did not describe the source of the control group. In the case group, the average age of hypertensive patients was between 42.58 and 68.05 years old. Moreover, only two included studies matched the age and gender proportions between control group and hypertensive group. The major testing method of *H*. *pylori* included enzyme linked immunosorbent assay (ELISA), ^13^C urea breath test (^13^C-UBT) and ^14^C urea breath test (^14^C-UBT) in all studies. The quality score of included articles were range from 3 to 8 according to the NOS and over fifty per cent of included studies were judged to be high-quality study (**S2 Table**).

**Table 1:** Major characteristics of included studies.

First author	year	country	study design	No. case	No. control	race	HDI^a^	mean age^b^	measure method	NOS score
Lip	1996	England	Case control	124	38	European population	developed country	53.2	ELISA	3
Kibria	2003	Italy	Case control	72	70	European population	developed country	53	^13^C-UBT	5
Shankar	2012	India	Case control	40	40	Asian population	developing country	46.71	ELISA	7
Wan	2018	China	Cross sectional	955	4213	Asian population	developing country	42.58	^13^C-UBT	7
Xiong	2020	China	Cross sectional	9638	7462	Asian population	developing country	68.05	^14^C-UBT	7
Liu	2007	China	Cross sectional	488	942	Asian population	developing country	51.65	ELISA	8

ELISA, enzyme-linked immunosorbent assay.

^13^C-UBT, 13C urea breath test.

^14^C-UBT,14C urea breath test. NOS, Newcastle–Ottawa Scale.

^a^HDI means human development index.

^b^mean age represents the average age of the case group.

### Risk of bias

Risk of bias in included studies were assessed via ROBINS-I tool (**S3 Table**). The majority of studies had controlled for one or more known confounding variables, such as age, gender, smoking and so on. However, none of included studies has fully considered all confounding factors. Thus, all studies presented a serious or moderate risk of bias in the confounding. Furthermore, one study had a moderate risk of bias in selection of participants. All studies had low risk of bias in intervention, deviations from intended interventions, missing data, measurement of outcomes and selection of the reported result.

### Meta‑analysis

**Fig 2:**
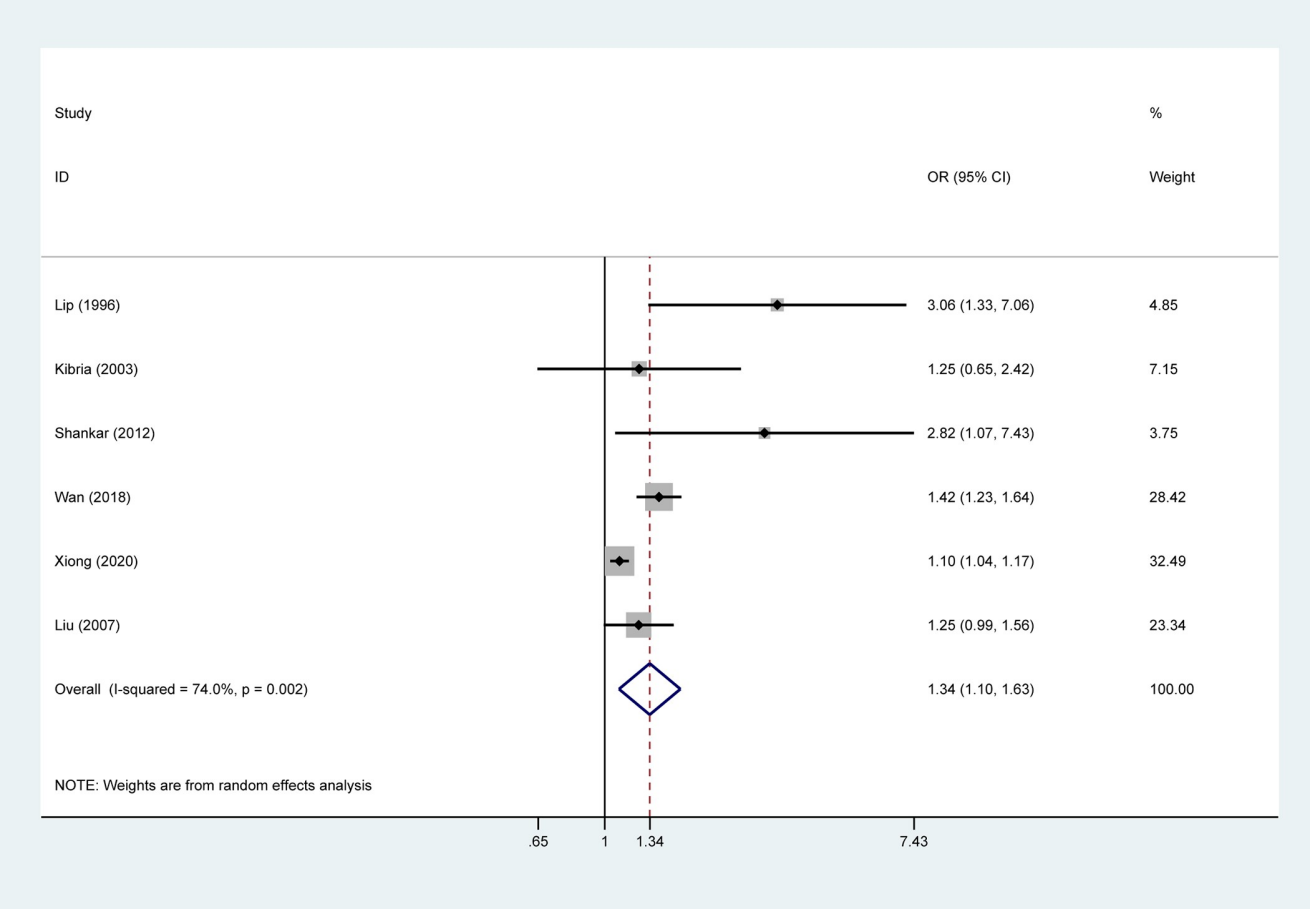
Forest plot of the pooled result between helicobacter pylori and hypertension.

The link of *H*. *pylori* infection with hypertension existed in three studies and their results with the ORs were range from 1.42 to 3.06. The meta‑analysis of all included studies suggested that *H*. *pylori* infection could significantly increase the risk of hypertension in the random-effects model (OR:1.34, 95% CI:1.10–1.63, P = 0.002, I^2^ = 74%) (**Fig 2**). The potential influence of confounding factors on the pooled result were performed by subgroup analysis according to the ethnicity (Asian population, European population), study design (case-control study, cross-sectional study), detection methods of *H*. *pylori* (ELISA,^13^C-UBT, ^14^C-UBT), study quality (low quality, high quality) and HDI (developed country, developing country). In subgroup analysis concerning on the study type, the pooled OR for case-control studies was 2.07 (95%CI:1.12–3.81) without significant heterogeneity (I^2^ = 41.6%, P = 0.181), while the pooled OR for cross-sectional studies was 1.24 (95%CI:1.03–1.48) with an evidence of heterogeneity (I^2^ = 81.3%, P = 0.005). By stratifying based on detection methods of *H*. *pylori*, a clear correlation between *H*. *pylori* and hypertension existed in the ^13^C-UBT subgroup without heterogeneity (OR:1.41, 95%CI:1.23–1.63, P = 0.709, I^2^ = 0%), yet no positive correlation was observed in ELISA subgroup (OR:1.97, 95%CI:0.99–3.91, P = 0.042, I^2^ = 68.5%). When subgroup analysis stratified by ethnicity and HDI, *H*. *pylori* could remarkably increase the risk of hypertension in both Asian population and developing country (OR:1.28, 95%CI:1.05–1.55, P = 0.003, I^2^ = 78.5%). However, no significant association was found in European population and developed country (OR:1.88, 95%CI:0.78–4.52, P = 0.099, I^2^ = 63.3%). In subgroup analysis concerning on study quality, high quality studies yielded a significant pooled OR of 1.28 (95%CI:1.05–1.55, P = 0.003, I^2^ = 78.5%). As the above results showed, detection methods of *H*. *pylori* and the study design may be the source of heterogeneity. The other variables could not significantly decreased the heterogeneity, suggesting that these variables could not explain the sources of heterogeneity.

### Sensitivity analysis and publication bias

**Fig 3:**
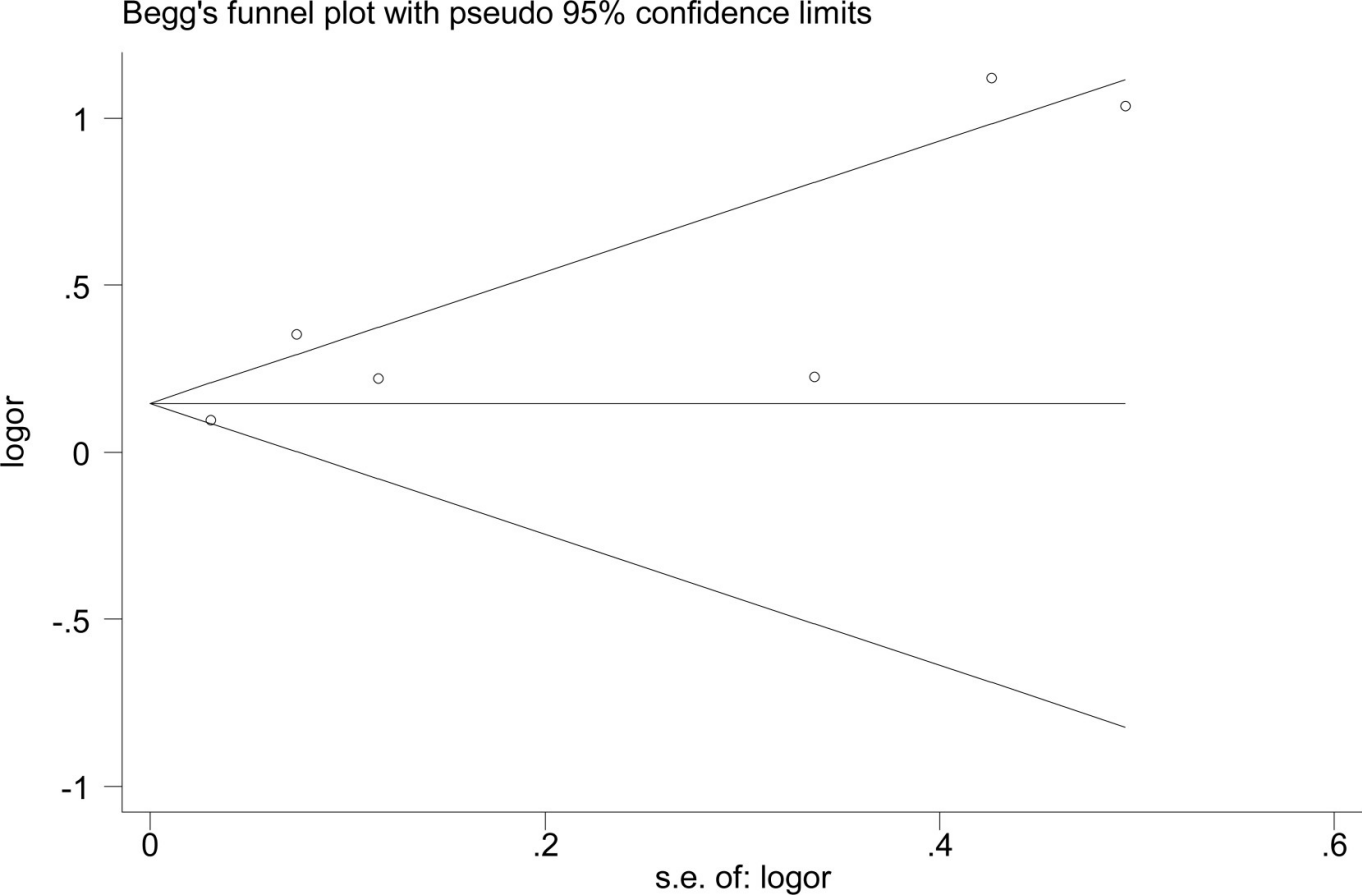
Funnel plot of helicobacter pylori and hypertension.

The sensitivity analysis was conducted to determine the stability of combined results by omitting one study each time. The results showed none of the studies influence the pooled result significantly, indicating that the pooled result was stable and reliable (**S4 Table**). Additionally, we detected the publication bias by two statistical methods (Egger test and Begg test) and neither method detected the publication bias (Egger test: p = 0.062, Begg test: p = 0.707) (**Fig 3**).

## Discussion

The influence of *H*. *pylori* on the hypertension has been examined in a great number of published studies, yet with inconsistent results. It is the first time to investigated on the correlation between *H*. *pylori* and hypertension through meta-analysis. This meta-analysis contain a larger sample size and applied rigorous statistical methods to get more reliable results. Combined results suggested that people with *H*. *pylori* infection had a 13.4% increased risk of hypertension. The positive relation with *H*. *pylori* and hypertension was also found in both Asian population and developing country. While, not seen to the similar result for European population and developed country. Compared with developed countries, the total infective rate of *H*. *pylori* was higher in developing countries. Especially, the most severely affected was Asian population [29, 30]. In addition, the high-salt eating habit of Asians could increase the colonization of *H*. *pylori* in the stomach [31]. The above reasons may explain the differences of pooled results between Asian population and European population. On the other hand, the economic environment might also potentially affect the pathogenesis of *H*. *pylori*. Compared with developing countries, a better health-care system in developed countries could timely reduce negative effects of *H*. *pylori*, including inflammatory reaction, toxins, dyslipidemia and so on. So as to further undermine the negative impacts of *H*. *pylori* in the development of hypertension.

The overall heterogeneity was high in this meta-analysis. We explored the source of heterogeneity by subgroup analysis. Notably, the heterogeneity was significantly reduced from 74% to 0% in the ^13^C-UBT subgroup and heterogeneity was still present in the ELISA subgroup. The above result indicated that the cause of heterogeneity may be explained by the difference in detection methods of *H*. *pylori*. The accuracy of serology experiments depends on the antigen used in ELISA kit. Thus, ELISA kits from different companies have different levels of diagnostic accuracy. In addition, the serologic detection could not precisely identify infection status. The ^13^C-UBT was recognized as a golden standard for detecting *H*. *pylori*. The ^13^C-UBT was superior to ELISA serology test in diagnosis of *H*. *pylori* infection because the level of *H*. *pylori* antibody is unpredictable [32, 33]. In addition, the heterogeneity also significantly decreased in the case-control studies subgroup (from 74% to 41.6%), however, there was still heterogeneity in the cross-sectional studies subgroup. On the one hand because the level of evidence in case-control studies was superior to cross-sectional studies. On the other hand, cross-sectional studies often collected samples from a specified population, which may cause potential biases.

The connection between *H*. *pylori* and hypertension has been proposed, according to most of the world’s clinical evidences correlating inflammation and salt intake with variation in arterial blood pressure [34, 35]. Inflammation may promote hypertension by causing endothelial dysfunction and inducing oxidative stress. Migneco et al have suggested that *H*. *pylori* infection may lead to the activation of the inflammatory cytokines cascade with the release of vasoactive substances from the site of infection [36]. A variety of inflammatory cytokines, including IL-1beta, IL-2, IL-6 and TNF-alpha, increased significantly in individuals with *H*. *pylori* infection [37–40]. Those inflammatory cytokines could promote insulin resistance [41]. Then, insulin resistance may further increase the total peripheral vascular tension [42]. In addition, people with *H*. *pylo*ri may enhance the level of fibrinogen, a biomarker of vascular inflammation which could suppress the decrease of nitric acid (NO), in turn, would cause vasoconstriction and increasing the peripheral blood vessel tension [43].

Salt intake was known as a major causative factor in hypertension. Studies indicated that *H*. *pylori* was strongly linked to high salt intake [44]. Beevers et al have certified that high salt consumption was directly related to *H*. *pylori* infection [12]. Akita is the region with the highest intake of salt in the daily diet, as well as the region with the highest *H*. *pylori* infection rate in Japan, which suggested a direct link between *H*. *pylori* and high sodium consumption [45]. The gastric mucosa could be injury by high salt, which enhanced the ability of *H*. *pylori* to survive and colonize in stomach [46]. These evidences might suggest that high sodium consumption and *H*. *pylori* in coordination with each other could enhanced the development of hypertension.

*H*. *pylori* directly interfered with the Vit D metabolism could be an alternate explanation for cause-and-effect link between *H*. *pylori* and hypertension. It has been confirmed that Vit D could regulate the Renin-Angiotensin-Aldosterone System (RAAS), a major hormonal mechanisms in the regulation of blood pressure [47]. *H*. *pylori*-related gastritis might interfere with absorption of multiple microelement and *H*. *pylori*-positive subjects had lower Vit D levels [13, 48]. Shafrir et al also proved that individuals without *H*. *pylori* infection could efficiently absorb Vit D in their diet [49]. It could be inferred that *H*. *pylori* was able to promote the development of hypertension by its effects on vitamin D metabolism in vivo.

It is the fast growth period that the number and the formation of the elder in the world in the 21st century and hypertension seriously threaten the health of the elderly. Controlling risk factors for hypertension actively could reduced cardiovascular diseases and better utilization of medical resources. So, reducing people’s exposure to risk factors of hypertension is an effective way to prevent and treat hypertension. At present, the hypertension guideline did not list *H*. *pylori* as the predisposing factor for hypertension and the indication for *H*. *pylori* eradication did not include hypertension. However, new discoveries had upended traditional ideas and indicated the underlying connection between hypertension and *H*. *pylori*. For hypertensive patients infected with *H*. *pylori*, their SBP and DBP both dropped significantly after the eradicating therapy for *H*. *pylori* [28]. Another study also confirmed the result that the 24-hours average systolic pressure and 24-hours average diastolic pressure in hypertensive patients dropped by 8.78 per cent and 19.04 per cent respectively after eradicating the *H*. *pylori* [36]. Another interesting study revealed that most hypertensive patients with *H*. *pylori* infection get their blood pressure down to normal and quit their medications after *H*. *pylori* eradication treatment [50]. Due to the global *H*. *pylori* pandemic in recent decades, the therapy of *H*. *pylori* eradication might be an alternative method for treatment or prevention of hypertension.

Though we conducted this meta-analysis rigorously, yet there still existed some limitations. First, hypertension is identified as a multifactorial disease and could be affected by the individual genetic background. Most studies came from Asian countries and no relevant studies in African countries. Thus, the results of this meta-analysis may not be appropriate for other ethnic groups. Second, all studies presented a serious or moderate risk of bias in the confounding and most research were not adequately adjusted for confounding factors (smoking, the situation of drugs, age, gender, family history of hypertension), which may have influenced the reliability of the evidence. Third, this meta-analysis consisted of cross-sectional and case–control studies, so the selection bias was inevitable and we may not be able to deduce the causal relationship between hypertension and *H*. *pylori* although it was etiologically plausible.

## Conclusions

This meta-analysis demonstrated that *H*. *pylori* could significantly increase the risk of hypertension, particularly among Asian population and developing country. Due to the widespread prevalence of *H*. *pylori* and the pathogenic behavior of *H*. *pylori* in hypertension, the *H*. *pylori* eradication may be an alternative method for treatment or prevention of hypertension. Moreover, additional high-quality research involving different regions and ethnicities is need to solve the key limitations in the meta-analysis.

## Supporting information

S1 Table PRISMA 2020 checklist. (DOCX)

S2 Table Quality evaluation for each included study by the Newcastle–Ottawa Scale. (DOCX)

S3 Table Evaluation of risk of bias for each included study by the ROBINS-I tool. (DOCX)

S4 Table Sensitivity analysis. (DOCX)
