# Correction: Association of hypertension with helicobacter pylori: A systematic review and meta‑analysis

**DOI:** 10.1371/journal.pone.0276919

**Published:** 2022-10-24

**Authors:** Yizhen Fang, Huabin Xie, Chunming Fan

Kibria et al. was incorrectly included as reference 28. As a result, all subsequent references are misnumbered. References 29–50 should be references 28–49. All occurrences of Kibria et al. should refer to Migneco et al. (previously reference 36, now reference 35).

As a result of the inclusion of the wrong reference there is an error in Table 1. The correct Table 1 can be found below.

**Table 1:** Major characteristics of included studies.

First author	year	country	study design	No. case	No. control	race	HDI^a^	mean age^b^	measure method	NOS score
Lip	1996	England	Case control	124	38	European population	developed country	53.2	ELISA	3
Migneco	2003	Italy	Case control	72	70	European population	developed country	53	^13^C-UBT	5
Shankar	2012	India	Case control	40	40	Asian population	developing country	46.71	ELISA	7
Wan	2018	China	Cross sectional	955	4213	Asian population	developing country	42.58	^13^C-UBT	7
Xiong	2020	China	Cross sectional	9638	7462	Asian population	developing country	68.05	^14^C-UBT	7
Liu	2007	China	Cross sectional	488	942	Asian population	developing country	51.65	ELISA	8

ELISA, enzyme-linked immuno sorbent assay.

^13^C-UBT, ^13^C urea breath test.

^14^C-UBT, ^14^C urea breath test. NOS, Newcastle–Ottawa Scale.

^a^HDI means human development index.

^b^mean age represents the average age of the case group.

There is an error in Fig 2. Please see the correct Fig 2 here.

**Fig 2:**
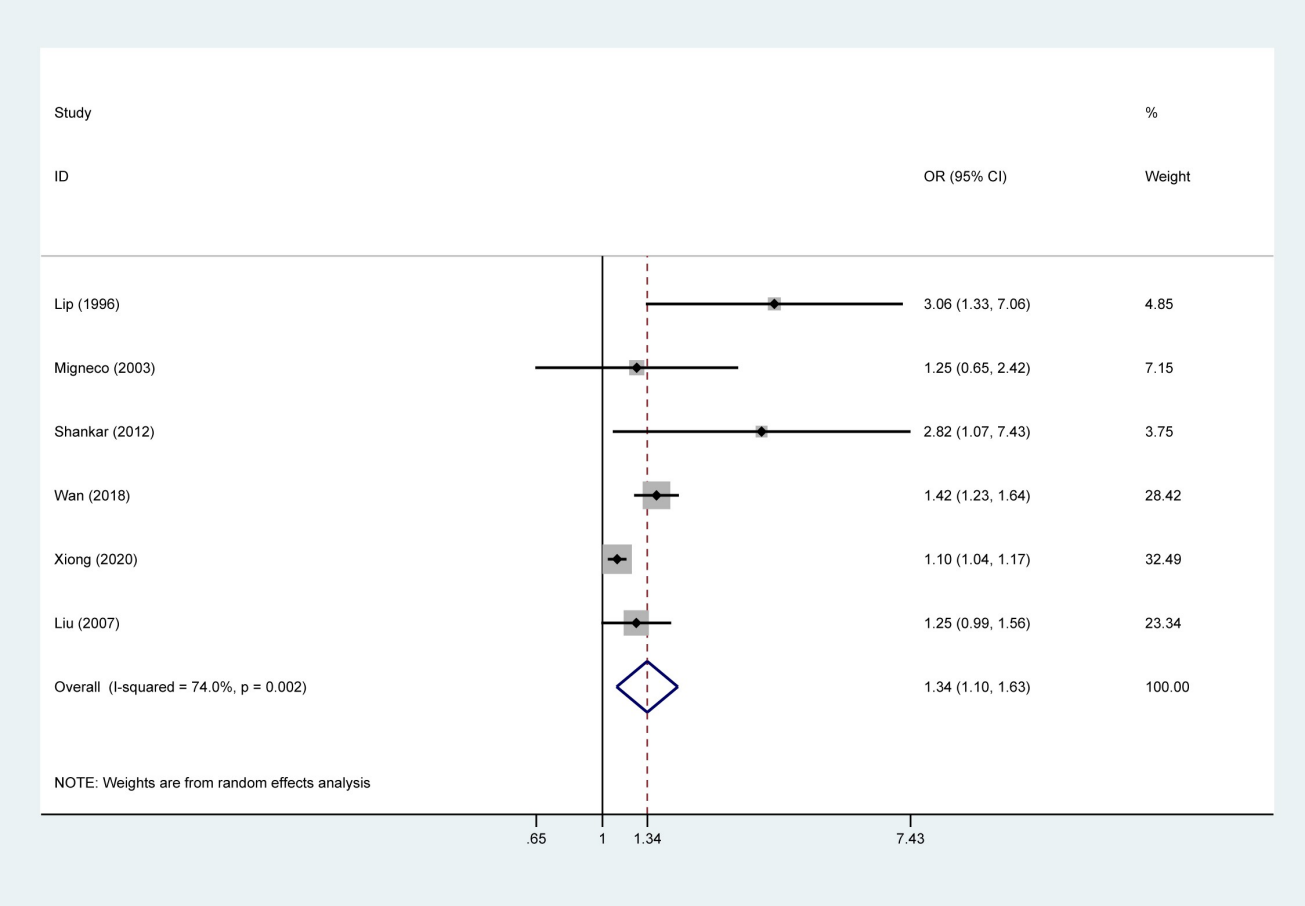
Forest plot of the pooled result between helicobacter pylori and hypertension.

There are errors in the Supporting Information files S2 Table, S3 Table, and S4 Table. Please view the correct files below.

## Supporting information

S2 Table Quality evaluation for each included study by the Newcastle–Ottawa Scale. (DOCX)

S3 Table Evaluation of risk of bias for each included study by the ROBINS-I tool. (DOCX)

S4 Table Sensitivity analysis. (DOCX)
