# Supplementary material for: Association of hypertension with helicobacter pylori: A systematic review and meta‑analysis
Source: PLoS One. 2022 May 19;17(5):e0268686. doi: 10.1371/journal.pone.0268686 (PMC9119435; doi:10.1371/journal.pone.0268686)
Supplement: S3 Table — (DOCX) [file pone.0268686.s003.docx]

S3 Table: Evaluation of risk of bias for each included study by the ROBINS-I tool

| First author | confounding | selection of participants | intervention | deviations from intended interventions | missing data | measurement of outcomes | selection of the reported result | Overall |
| --- | --- | --- | --- | --- | --- | --- | --- | --- |
| Lip | Serious | Moderate | low | low | low | low | low | Serious |
| Kibria | Moderate | low | low | low | low | low | low | Moderate |
| Shankar | Moderate | low | low | low | low | low | low | Moderate |
| Wan | Moderate | low | low | low | low | low | low | Moderate |
| Xiong | Moderate | low | low | low | low | low | low | Moderate |
| Liu | Moderate | low | low | low | low | low | low | Moderate |
