# Supplementary material for: Correction: Association of hypertension with helicobacter pylori: A systematic review and meta‑analysis
Source: PLoS One. 2022 Oct 24;17(10):e0276919. doi: 10.1371/journal.pone.0276919 (PMC9591046; doi:10.1371/journal.pone.0276919)
Supplement: S2 Table — (DOCX) [file pone.0276919.s001.docx]

**S2 Table. Quality evaluation for each included study by the Newcastle–Ottawa Scale**

|  | **Selection** | | | | **Comparability** | **Outcome** | | |
| --- | --- | --- | --- | --- | --- | --- | --- | --- |
|  | Case definition with  independent validation | Representativeness of the cases | Selection of controls | Definition of controls | Comparability of cases and cohorts on the basis of the design or analysis | Ascertainment of exposure | Same method of ascertainment for cases and controls | Non-Response rate |
| Lip et al, 1996 | — | — | — | — | — | ★ | ★ | ★ |
| Migneco et al, 2003 | — | — | — | ★ | ★ | ★ | ★ | ★ |
| Shankar et al, 2012 | ★ | ★ | — | ★ | ★ | ★ | ★ | ★ |
| Wan et al, 2018 | ★ | ★ | — | ★ | — | ★★ | ★ | ★ |
| Xiong et al, 2020 | ★ | ★ | — | ★ | — | ★★ | ★ | ★ |
| Liu et al, 2007 | ★ | ★ | ★ | ★ | — | ★★ | ★ | ★ |
