# Supplementary material for: Correction: Association of hypertension with helicobacter pylori: A systematic review and meta‑analysis
Source: PLoS One. 2022 Oct 24;17(10):e0276919. doi: 10.1371/journal.pone.0276919 (PMC9591046; doi:10.1371/journal.pone.0276919)
Supplement: S4 Table — (DOCX) [file pone.0276919.s003.docx]

**S4 Table. Sensitivity analysis**

| **Excluding study** | **OR (95% CI)** |
| --- | --- |
| None | 1.34 (1.10-1.63) |
| Lip et al, 1996 | 1.27 (1.06-1.52) |
| Migneco et al, 2003 | 1.35 (1.09-1.67) |
| Shankar et al, 2012 | 1.29 (1.06-1.56) |
| Wan et al, 2018 | 1.32 (1.03-1.69) |
| Xiong et al, 2020 | 1.44 (1.17-1.77) |
| Liu et al, 2007 | 1.40 (1.08-1.81) |
